# Supplementary material for: High-Throughput Functional Analysis of CFTR and Other Apically Localized Proteins in iPSC-Derived Human Intestinal Organoids
Source: Cells. 2021 Dec 4;10(12):3419. doi: 10.3390/cells10123419 (PMC8699884; doi:10.3390/cells10123419)
Supplement: Supplementary file 1 [file cells-10-03419-s001.zip › cells-1444336-supplementary.pdf]

Supplementary Figures

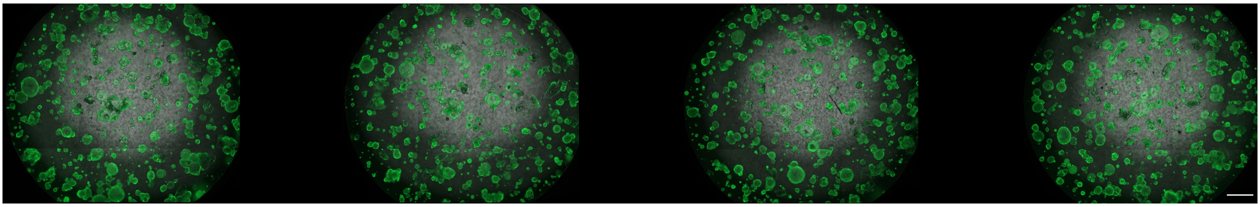

Figure S1: *Opened* organoid for functional analysis of ion channels. Merged bright field and fluorescence of image of *opened* organoids in multi-well plates depicting homogenous organoid density across wells. Organoids were loaded with a live cell viability marker, Calcein-Am. Scale bar = 500 μm

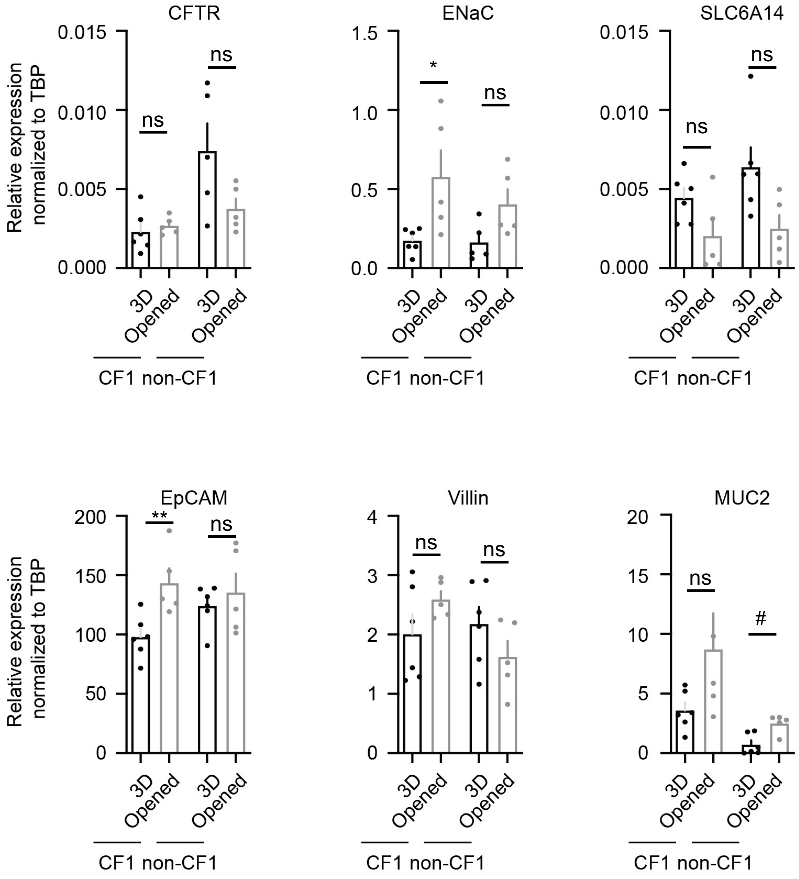

Figure S2: Gene expression studies on iPS derived HIOs. Expression of intestinal apical membrane ion channels (CFTR and ENaC), amino acid transporter (SLC6A14), epithelial cell marker (EpCAM), intestinal epithelial cell marker (Villin) and goblet cells marker (MUC2), relative to house keeping gene *TBP*, in CF and MC organoids in 3D and opened formats using RT-qPCR. Bar graph and error bars depict mean ± SEM. Each dot represents an independent biological replicate and each biological replicate = independent organoid passage. Unpaired t-test was performed between 3D and *opened* organoids, ns indicates no significant changes in gene expression (* P = 0.0329, ** P = 0.0097, $^{\#}$ P = 0.0066).

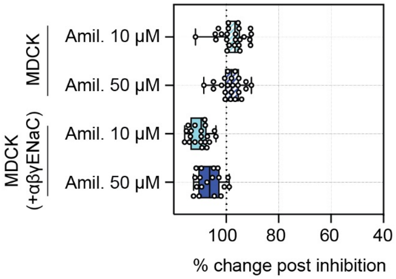

Figure S3: Functional validation of ENaC activity measured in MDCK cells. Box and whisker plot of parental MDCK control cells or MDCK cells expressing αβγENaC acutely treated with amiloride (10 μM or 50 μM) in presence and absence of 140 mM extracellular sodium (**** $P < 0.001$, $n > 3$ biological replicates, $n = 3$ technical replicates). Box plot depicts the median value and bounds depict IQR ranges with the whiskers defining the minima and maxima values.

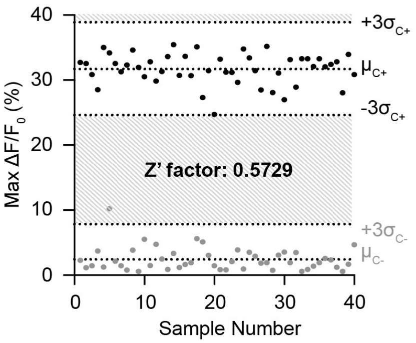

Figure S4: Functional validation of ENaC inhibition in *opened* non-CF1 organoids. Non-CF *opened* organoids were acutely treated with amiloride (50 μM) or vehicle control DMSO. Bland-Altman Plot depicting the reproducibility of ENaC inhibition with amiloride treatment. Each point represents measurement from and individual well of a 96 well plate. Black points indicate maximum change in fluorescence measurement with acute amiloride treatment in comparison to grey points indicate DMSO control treatment.
